# Eco-epidemiological and pathological features of wildlife mortality events related to cyanobacterial bio-intoxication in the Kruger National Park, South Africa

**DOI:** 10.4102/jsava.v87i1.1391

**Published:** 2016-10-31

**Authors:** Roy Bengis, Danny Govender, Emily Lane, Jan Myburgh, Paul Oberholster, Peter Buss, Leon Prozesky, Dewald Keet

**Affiliations:** 1 Private Wildlife Practice, Port Alfred, South Africa; 2 Scientific Services, South African National Parks, Skukuza, South Africa; 3 Department of Paraclinical Sciences, University of Pretoria, Onderstepoort, South Africa; 4 Department of Research and Scientific Services, National Zoological Gardens of South Africa, South Africa; 5 CSIR Natural Resources and the Environment, Stellenbosch, South Africa; 6 Veterinary Wildlife Services, South African National Parks, Skukuza, South Africa; 7 Private Wildlife Practice, Phalaborwa, South Africa

## Abstract

Over the past decade, several clustered, multispecies, wildlife mortality events occurred in the vicinity of two man-made earthen dams in the southern and south central regions of the Kruger National Park, South Africa. On field investigation, heavy cyanobacterial blooms were visible in these impoundments and analysis of water samples showed the dominance of *Microcystis* spp. (probably *Microcystis aeruginosa*). Macroscopic lesions seen at necropsy and histopathological lesions were compatible with a diagnosis of cyanobacterial intoxication. Laboratory toxicity tests and assays also confirmed the presence of significant levels of microcystins in water from the two dams. These outbreaks occurred during the dry autumn and early winter seasons when water levels in these dams were dropping, and a common feature was that all the affected dams were supporting a large number of hippopotamuses (*Hippopotamus amphibius*). It is hypothesised that hippopotamus’ urine and faeces, together with agitation of the sediments, significantly contributed to internal loading of phosphates and nitrogen – leading to eutrophication of the water in these impoundments and subsequent cyanobacterial blooms. A major cause for concern was that a number of white rhinoceros (*Ceratotherium simum*) were amongst the victims of these bio-intoxication events. This publication discusses the eco-epidemiology and pathology of these clustered mortalities, as well as the management options considered and eventually used to address the problem.

## Introduction

Cyanobacteria are considered to be one of earth’s earliest photosynthetic organisms (Bell & Coombe 1970). They are not true algae but belong to a group of ancient prokaryotic bacteria. Cyanobacteria occur commonly in low numbers in most aquatic environments; however, during times of environmental disturbances that result in eutrophication (nutrient over-enrichment) of these aquatic systems, cyanobacteria may gain a selective advantage over other resident zoo- and phytoplankton populations and increase in both numbers and bio-volume (Oliver & Ganf 2000; Paerl & Huismans 2008). Paerl and Huismans (2008) also reported that cyanobacteria generally grow better at higher temperatures (often > 25 °C) than other phytoplankton species, which gives them a competitive advantage. The resultant ‘bloom’ can be seen as a dense layer of green suspension in the water column, and at times as a green scum layer on the water surface.

Eutrophication is a natural ageing process of lakes and man-made dams. It is characterised by a geologically slow shift from in–lake biological production driven by allochthonous loading of nutrients (nutrients from outside the aquatic system), to production driven by autochthonous processes (nutrients from within the aquatic system). This typically slow process over centuries can be greatly accelerated to decades by human intervention in the natural biogeochemical cycling of nutrients within a watershed (Rast & Thornton 1996). Eutrophication is frequently caused by the addition of nutrients from human activities, in this context called anthropogenic eutrophication. The phenomenon is usually a consequence of intensive agricultural and industrial activities and the development of large sewage systems associated with large metropolitan areas (Harding & Paxton 2001; Van Halderen, Harding & Wessels 1995). In many cases the direct consequences of eutrophication are the excessive growth of autotrophic organisms. In standing waters, this generally manifests in blooms of undesirable cyanobacteria.

Cyanobacteria blooms are generally not a feature of flowing water systems because the nutrient load and the cyanobacterial cells are continuously diluted and dispersed. However, in southern Africa, bloom formation is commonly seen in man-made dams that have received organic material from livestock enterprises or dysfunctional sewage treatment plants, and/or run-off agricultural fertilisers (Clarke & Clarke 1967; Gous 1999; Oberholster *et al*. 2013; Steyn 1949). Theory suggests that these shallow lake systems can exist in one of two states, either a plant dominated, clear water state, or a turbid, algal dominated state (Scheffer *et al*. 1993; Scheffer & Van Nes 2007). Phosphate availability in the water column is the primary factor driving the switch. Under eutrophic conditions, phytoplankton (including cyanobacteria) proliferates, further increasing turbidity and decreasing the light availability at the lake bottom, causing the system to shift into the algal dominated state.

Although there are a few previous studies that indicate eutrophication may be caused by animals, fish and birds in freshwater bodies (Bomchul *et al*. 2001; Oberholster *et al*. 2009a), little is known about eutrophication caused by the influence of mega-herbivores and their excreta in aquatic ecosystems.

Certain species of cyanobacteria are capable of producing highly toxic substances. Toxicoses associated with cyanobacterial blooms have been recorded in wildlife, domestic mammals, waterfowl and fish in South Africa (Clarke & Clarke 1967; Kellerman *et al*. 2005; Oberholster *et al*. 2009b; Steyn 1949). However, not all toxigenic species or blooms of cyanobacteria will always be toxic to animals, as rapid changes in cyanobacterial biomass and toxin content can occur over short periods of time (Jiang *et al*. 2008; Oberholster *et al*. 2005; Wicks & Thiel 1990). The identification of a cyanobacterial genus by microscopic morphology does not necessarily indicate its potential for toxin production. Different strains of one species can be morphologically identical but differ in toxigenicity. For example, *Microcystis aeruginosa*, which is the most abundant toxic cyanobacterial genus in South Africa, may contain both toxic and nontoxic strains (Harding & Paxton 2001; Oberholster *et al*. 2005).

Other factors that might play a role are variation in susceptibility of different animal species, dosage exposure rates, the age and sex of animals, type of digestive system (ruminant, monogastric or hindgut digesters), and the amount of food in an animal’s gastro-intestinal system. The situation in the man-made earthen dams in Kruger National Park (KNP), South Africa appears to be unique with both shallow lake dynamics and high hippopotamus numbers driving eutrophication through urinary and faecal inputs as well as bringing those nutrients into solution because of physical activity by hippopotami at the lake bottom (Bengis, Govender & Keet 2008; Buss & Bengis 2012). In this study, the eco-epidemiology and pathology of wildlife mortalities caused by several toxigenic *Microcystis* blooms in man-made dams are discussed.

## Study area and background

**FIGURE 1:**
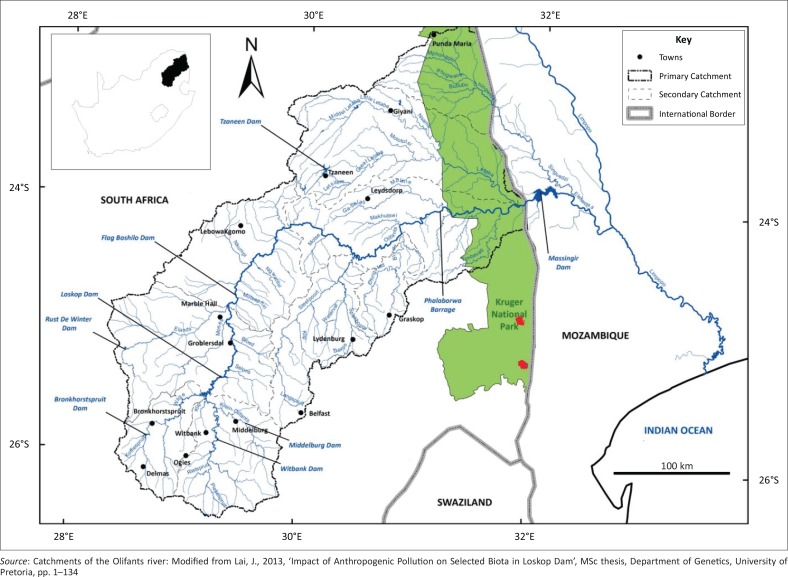
Map of the Kruger National Park indicated in green, with the locality of the two affected dams in red.

The KNP is one of the largest conservation areas in Africa and covers 19 485 km^2^ on South Africa’s eastern border with Mozambique (Figure 1).

In the 1960s and 1970s, following severe droughts in the KNP and with natural flow in the major perennial rivers being depleted by upstream extraction for industry, agriculture and forestry, a management decision was made to attempt to stabilise surface water in the Park. The sinking of localised boreholes, as well as the damming of minor and major seasonal drainages and perennial rivers, accomplished this objective (Pienaar 2010).

In addition, a decision was made, during periods of drought to lethally manage certain dominant mega-herbivore species such as elephants (*Loxodonta africana*), buffaloes (*Syncerus caffer*) and hippopotami (*Hippopotamus amphibius*), to reduce intra-specific competition and competition with less abundant and less competitive herbivores and prevent rangeland degradation (Pienaar 2010). From 1968–1994, excess hippopotami, based on an annual aerial census, were removed lethally or non-lethally from the river systems. In 1993, a severe drought resulted in a significant reduction in hippopotamus numbers, indicating that populations may be naturally regulated, and further lethal management was curtailed in 1995. Thereafter followed a decade of above average annual rainfall, hippopotamus numbers increased dramatically and their populations flourished, causing dispersal of significant numbers of hippopotami out of larger river systems into smaller water bodies. This migration of hippopotami was destined to have a profound impact on these smaller aquatic systems.

## Sequence of events and results

**FIGURE 2:**
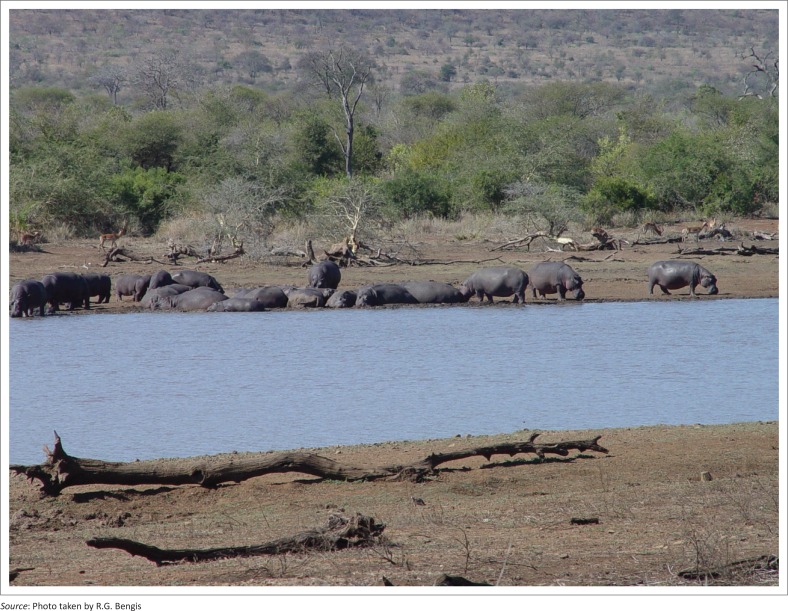
High concentrations of hippopotami at Nhlanganzwane Dam.

In 2005, following a very dry late-summer season and an abnormally warm autumn and early winter, the water level in the Nhlanganzwane Dam (31°58’27” E, 25°13’52” S) was dropping, while nevertheless still supporting a large resident hippopotamus population (Figure 2).

Between March and June of that year, a cluster of mortalities involving several large mammalian species was noted in the vicinity of this dam. Most carcasses were already several days old and in an advanced stage of decomposition, or heavily utilised by vultures and other scavengers at the time of the investigation. Blood smears and tissue specimens were taken from several of these carcasses and were found to be negative for *Bacillus anthracis* (anthrax) by microscopy and culture.

### Macro- and histopathology

**FIGURE 3:**
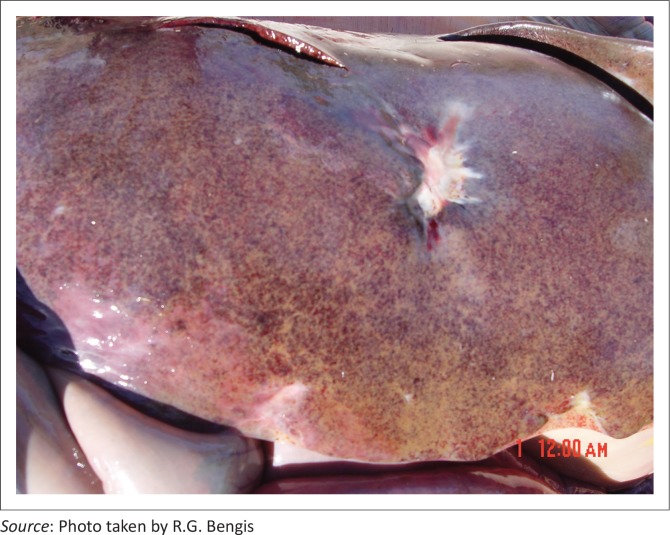
Macroscopic appearance of zebra liver following death from cyanobacterial intoxication.

**FIGURE 4:**
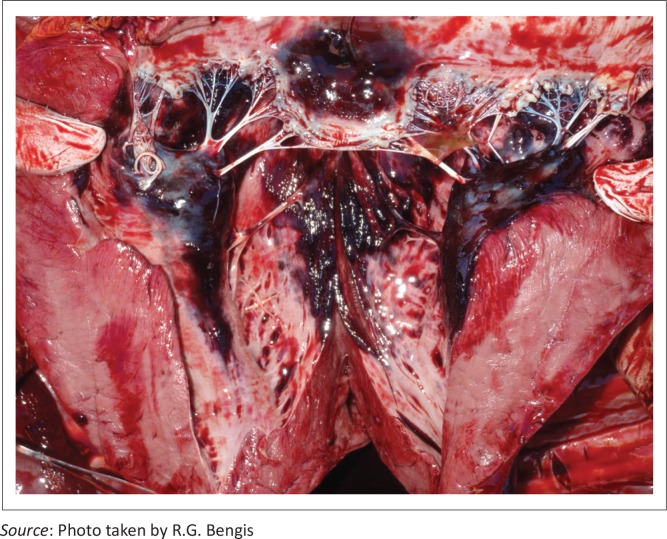
Diffuse subendocardial ecchymoses in a zebra following cyanobacterial intoxication.

**FIGURE 5:**
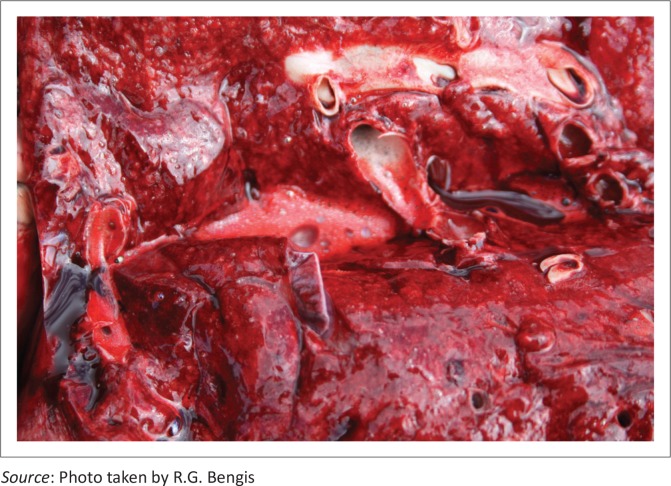
Severe pulmonary oedema in a zebra following cyanobacterial intoxication.

Several species investigated showed similar macro- and histopathological lesions. Zebra (*Equus burchelli*) carcasses showed severe hepatic swelling with mottled orange and brown discolouration (Figure 3), and multiple haemorrhages (petechiae, ecchymoses and suggulations) present beneath abdominal and thoracic serosal surfaces, sub-epi and endocardial tissues (Figure 4) and in subcutaneous tissues and fascial planes. The carcasses were also icteric and there was severe pulmonary oedema with froth in the major airways (Figure 5).

Histologically, zebra liver sections showed massive hepatic congestion, haemorrhage, necrosis and apoptosis as well as moderate post-mortem change and putrefaction. Hepatic sinusoids were markedly distended with blood, separating and surrounding individual disassociated or small rafts of hepatocytes, small numbers of which contained dark basophilic chromatin remnants. In addition, severe acute diffuse pulmonary oedema and alveolar emphysema, moderate acute renal congestion and marked splenic congestion were present in zebra.

In blue wildebeest (*Connochaetes taurinus*) carcasses, unclotted blood or foam was seen draining from the nose and blood-tinged fluid was often present in the thorax and abdomen. Multiple acute small to extensive haemorrhages were variably present in the subcutaneous tissues, fascial planes, serosal surfaces, lung tissue surrounding the airways, internal fat stores, small intestinal wall, musculature, lymph nodes, spleen and, less commonly, kidney and adrenal. Livers were generally enlarged, mottled orange or brown and swollen with rounded edges and localised areas of petechiae and echymoses under the Glisson’s capsule. On cut surface liver parenchyma was very friable with variably exaggerated lobulation patterns.

In addition, extensive acute subepicardial petechiae and ecchymoses occurred on both atria, coronary groove adipose tissue and ventricles and severe subendocardial ecchymoses and suggulations mainly affected the papillary muscles. There were multifocal subpleural haemorrhages and haemorrhages in the proximity of the main stem bronchi were common. Three blue wildebeest had large areas (10 cm × 12 cm diameter) of acute pulmonary parenchymal haemorrhage. There was also severe acute, diffuse pulmonary congestion and oedema characterised by moist lungs with clear watery to blood-tinged fluid draining from cut surfaces and white froth in the main airways. Multiple acute ecchymotic haemorrhages occurred in the tracheal mucosa of some animals. Variable alveolar emphysema was also present.

In the liver sections of the blue wildebeest, hepatic sinusoids were markedly congested, often separating and surrounding individual disassociated or small rafts of hepatocytes. Hepatocytes showed variable hydropic degeneration and lipidosis. Occasionally individually necrotic and/or apoptotic cells with dark basophilic chromatin remnants were present. More severe cases showed extensive necrosis of the periacinar to mid-zonal hepatocytes characterised by cell swelling and fragmentation, karyolysis, apoptosis, and karyorrhexis, with extensive sinusoidal congestion and haemorrhage. Often, only a narrow rim of degenerative hepatocytes surrounded the portal triads and virtually no intact hepatocytes were present.

In white rhinoceroses, there was unclotted blood bubbling from the nasal orifices. On removing the skin panels, numerous haemorrhages varying from petechiae to suggulations were observed involving the subcutaneous tissues, muscle masses and fascial planes. There was a yellowish discolouration surrounding some of these haemorrhages. There was approximately 2 L of blood-tinged fluid in the abdominal cavity, and the gastro-intestinal tract was well filled.

The spleen had multiple punctate haemorrhages under the capsule, and there were haemorrhages in most lymph nodes. The liver was grossly enlarged with rounded margins, extremely friable and dark reddish brown in colour. The hepatic parenchyma was dark, soft and jelly-like with gross loss of macro-visible architecture. The lungs were moist with areas of haemorrhage and areas of atelectasis, but there was no foam in the airways. There were numerous haemorrhages in the pleura and mediastinum. In the heart, there was acute diffuse haemorrhaging in the sub-epicardial as well as the subendocardial tissues of both ventricles. In the abdominal cavity, there were multifocal haemorrhages present under the peritoneum, in the omentum and the mesenterium.

In the liver sections of white rhinoceros (*Ceratotherium simum*), hepatic architecture was extensively disrupted by congestion, haemorrhage and massive hepatocyte necrosis affecting hepatocytes of all lobular zones. Rare apoptotic hepatocytes were also present. Associated histological findings included marked acute focal adrenal cortical and medullary congestion and haemorrhage; marked acute diffuse pulmonary congestion and protein-rich alveolar oedema with discrete foci of atelectasis; mild acute multifocal renal interstitial haemorrhage with scattered tubules filled with protein-rich fluid, and moderate acute focal subepicardial, subendocardial and myocardial haemorrhage.

The macro- and histopathological lesions seen in impalas (*Aepyceros melampus*) were similar to those described for wildebeest. These macro- and histopathological lesions are suggestive of a diagnosis of *Microcystis* intoxication.

**FIGURE 6:**
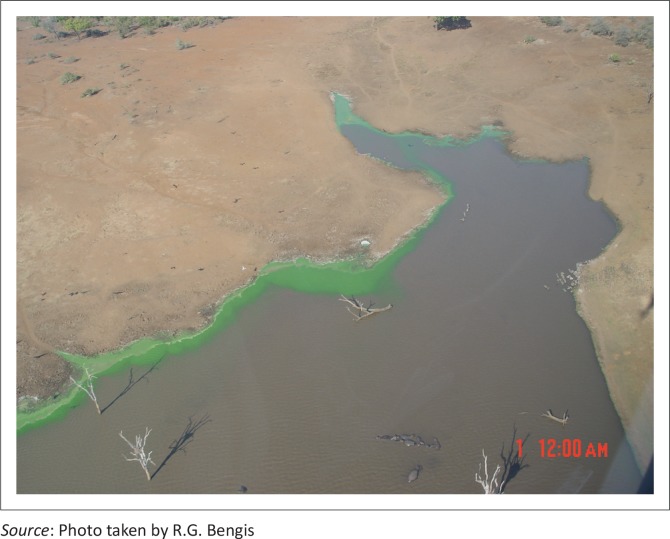
Aerial view of wind-driven algal rafting at Nhlanganzwane Dam.

**FIGURE 7:**
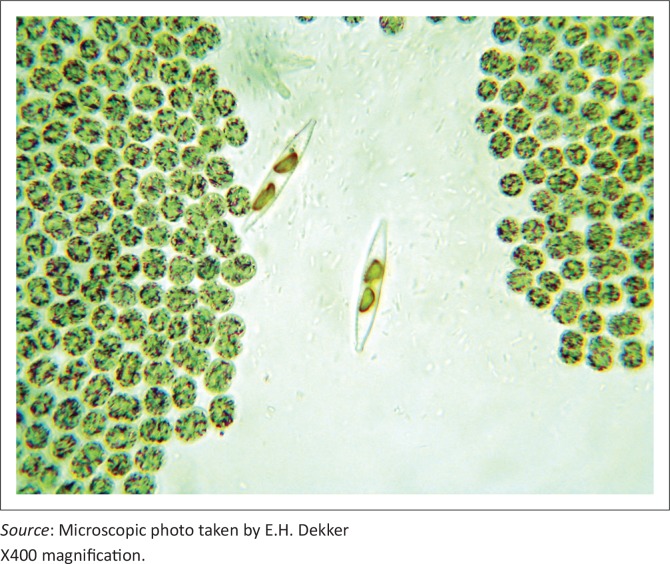
Microscopic image showing sheets of *Microcystis* spp. organisms plus two diatoms.

These findings triggered an investigation of this dam, and a severe cyanobacterial (blue-green algal) bloom was detected in the water column, with some rafting of green scum on the down-wind side (Figure 6). Sub-surface grab water samples (1 L) for analysis of the phytoplankton population structure were collected. All algal identifications were made with a compound microscope at 1250 x magnification (Van Vuuren *et al*. 2006). Strip counts were made until at least 300 individuals of each of the dominant phytoplankton species had been counted. Microscopic examination showed a dominance of a *Microcystis* species, most probably *M. aeruginosa* (Figure 7). Mouse toxicity assays and follow-up histopathology on the mouse organs supported the early presumptive diagnosis of *Microcystis* intoxication.

From the analysis of the ranger’s diary and follow-up field investigations, a total of 54 carcasses were detected, most of which were spatially clustered around this dam and included ruminants, hindgut fermenters and predators. Most carcasses were either too decomposed for diagnostic purposes or had been ‘hollowed out’ by vultures, but it was assumed from the carcass spatial distribution, condition and species mix (which included 23 wildebeest , 9 zebras, 7 white rhinoceroses, 2 lions [*Panthera leo*] and 2 cheetahs [*Acinonyx jubatus*]) that these mortalities could be attributed to a point source cyanobacterial intoxication. There were also no other abnormal mortality events detected or reported elsewhere in the KNP during the same time.

Control options were evaluated, as there was concern regarding populations of white rhinoceros and the rare wild dogs (*Lycaon pictus*) in the immediate vicinity of this dam. While evaluating the various control options, a rangeland fire burned most of the grazing in the vicinity of this dam. This situation forced the hippopotami to disperse from the dam and move to the Sabie and Crocodile River systems. Visible algal blooms appeared to subside over the following weeks and, as the burned area was unattractive to other herbivores because of lack of grazing, no further mortalities were recorded after early July.

Two years later, during June 2007 a new cluster of mortalities was detected in the same geographical area. The Nhlanganzwane Dam once again hosted a large resident hippopotamus population and a significant cyanobacterial bloom was present. The dominance of *Microcystis* sp. was again demonstrated microscopically in water samples from this dam. A commercial Abraxis -microcystin ELISA kit (Aqualytic, Environmental and Laboratory Services) and a catfish hepatocyte culture bio-assay (Masango *et al*. 2010; Naicker, Myburgh & Botha 2007) demonstrated high levels of biotoxins (103 mg/L – 124 mg/L versus control samples from unaffected dams of 0.001 mg/L – 1.1 mg/L). A diagnosis of cyanobacterial bio-intoxication was confirmed at post-mortem examination when typical macroscopic lesions were observed in fresh zebra and wildebeest carcasses. Histopathological lesions, although non-specific, were similar in all species and consistent with those reported for microcystin toxicity (Hooser 2000; Kellerman *et al*. 2005).

During one necropsy, a male zebra foetus (towards the end of term) was found to have an enlarged and discoloured liver, which microscopically showed similar lesions associated with cyanobacterial intoxication, suggesting transplacental intoxication with microcystins.

**FIGURE 8:**
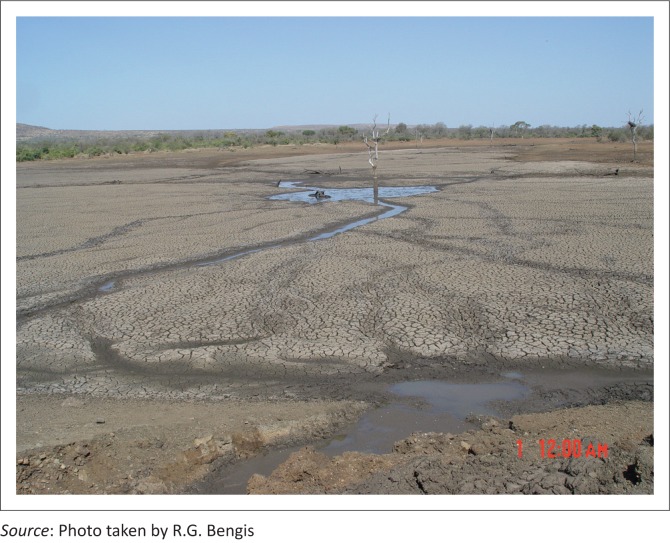
Hippopotamus dung-ladened sediment, Nhlanganzwane Dam.

During this second mortality event, the carcasses of nine white rhinoceros, ten zebras and ten wildebeest were found in the vicinity of Nhlanganzwane Dam. In August 2007, the decision was made to breach the wall of this dam and drain it, forcing the hippopotami to disperse to other water bodies. This decision was in line with the current KNP water provision policy, which is to mimic natural surface water distribution and remove redundant water points. The bottom of the dam was found to be covered with a tenacious and odiferous organic sediment layer, in excess of one metre deep in places, of which hippopotamus dung formed a significant component (Figure 8). Following the drainage of this dam, no further mortalities associated with cyanobacterial intoxication were recorded in that geographical area.

**FIGURE 9:**
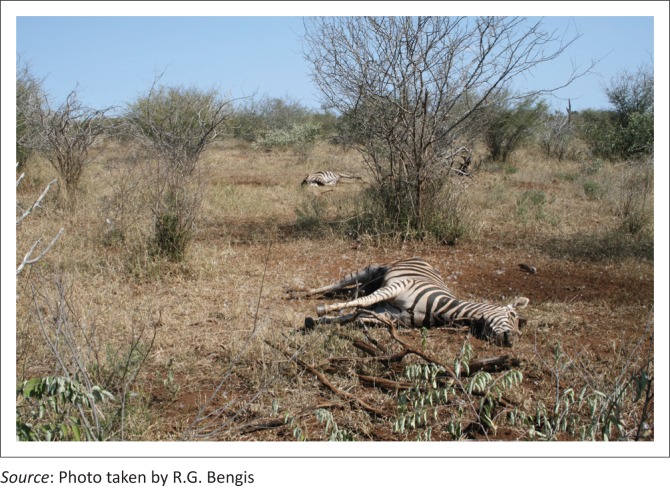
Zebra mortalities – Shilolweni Dam.

In April, 2008, a third mortality incident involving mainly zebras, wildebeest, impalas and white rhinoceroses was detected in the vicinity of Shilolweni Dam (31°50’00” E; 24°49’28” S), approximately 50 km north of the previous events described. Once again, there was a significant hippopotamus presence – approximately 70 animals (Figure 9).

**FIGURE 10:**
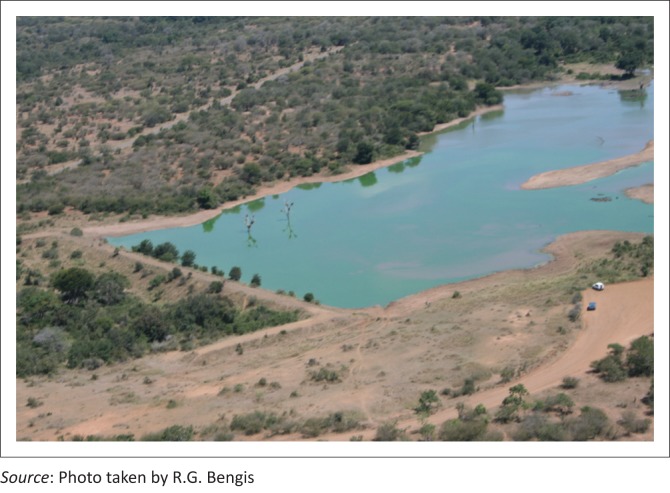
Aerial view of algal bloom at Shilolweni Dam.

Blood smears stained with Giemsa and tissue samples cultured on P.L.E.T. agar were negative for anthrax, and necropsies on fresh carcasses demonstrated characteristic hepatic lesions with generalised haemorrhages and pulmonary oedema consistent with *Microcystis* intoxication. Histopathology confirmed the presence of typical microscopic lesions. Aerial surveys of the area showed that only Shilolweni Dam was affected, and a massive blue-green algal bloom was apparent (Figure 10).

This dam was also an important and popular viewing site for tourists and KNP management dealt with this bio-intoxication event by pumping the dam dry. Hippopotami and other resident and migratory species were forced to move away from this water source, and no further mortalities were recorded. To salvage this dam, the nutrient-rich sediment needed to be scoured from the exposed dam floor before the summer rains arrived. This was attempted using heavy earth moving equipment, but because of the depth and tenacity of the sediment only one half could be removed before the summer rainy season started.

In 2009, the dam was partially filled by the summer rain, and a few hippopotami returned, but no mortalities were recorded. By July 2010, the dam had refilled completely and once again supported a high density of hippopotami. It was at this time that a second significant clustered mortality event occurred at this impoundment, affecting mainly zebras and wildebeest migrating through this area, as well as a few resident white rhinoceroses. A total of 17 zebra, 7 wildebeest and 3 white rhinoceros carcasses were detected. The dam wall was breached in September, 2010, as a permanent solution, after which no further associated mortalities were recorded.

## Discussion

The management policy regarding stabilisation of surface water resulted in the building of many small dams across seasonal drainages in the KNP during the 1950s and 1960s. These man-made impoundments matured over the ensuing 40 years and became important wildlife viewing sites for tourists. An unfortunate characteristic of many of these small impoundments, because of their structure and water inflow dynamics, is that they act as closed shallow lake systems. These characteristically trap nutrient-rich sediment and, because they are shallow and have thermal mixing of the water column with light penetration to the bottom, provide a situation that supports phytoplankton proliferation. This process also appeared to be accelerated by the influx of significant numbers of hippopotami.

**FIGURE 11:**
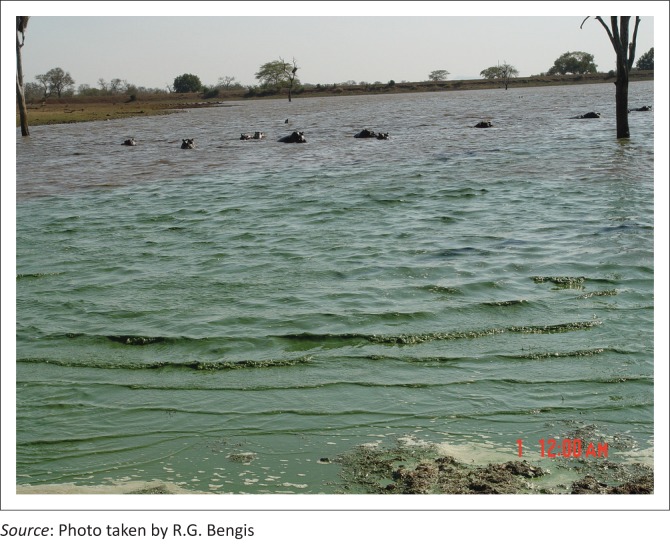
Hippopotami and algal bloom at Shilolweni Dam.

At the time of the cyanobacterial bloom events, Nhlanganzwane Dam had a water surface area of approximately 15 hectares, and supported a resident hippopotamus population of in excess of 100 animals, while Shilolweni Dam with a surface area of 12 hectares had a resident population of approximately 70 hippopotami. Shrinking water volumes in the dry, early winter season, together with unusually high hippopotamus densities, are believed to have resulted in progressive eutrophication of these still water bodies, with large amounts of nitrogenous and phosphate compounds accumulating in the sediments because of urinary and faecal deposits by the resident hippopotami. The environmental temperatures ranged from 24 °C – 35 °C and water temperatures were in the mid-20s. In addition, internal loading through the physical activities of the hippopotami is thought to agitate and stir up the sediment, placing even more of these nutrients into the water column and increasing their availability to the cyanobacteria, similar to the results of the activities of bottom-feeding fish in other systems (Figure 11).

It is interesting to note that the four mortality events reported above were both spatially (vicinity of affected dams) and temporally (late autumn or early winter) clustered. On a finer time scale, the incidence of new mortalities was also temporally clustered, and probably related to the wind direction and the rafting of algal scum to preferred drinking sites on the perimeter of the dams.

Another interesting observation was that in relation to their population estimates in the vicinity of these dams, white rhinoceroses, zebras and wildebeest were over-represented in the carcass counts, whereas no elephant, buffalo or hippo carcasses were found, even though these species were common in the area.

A hypothesis to explain this observation is that elephants, hippopotami and buffaloes are species that wade into the water to drink away from the water’s edge, and can thus avoid the algal ‘scum line’ that has rafted onto the shoreline. In addition, elephants, buffalo herds and hippopotami will approach a water point from any direction. White rhinoceroses, zebras and wildebeest are generally non-wading species, and usually approach the water point from the ‘down-wind’ side in order to scent ambushing predators (Hayward & Hayward 2012). These species are therefore more likely to drink from the shoreline contaminated with high levels of cyanobacteria in the rafting scum line.

From the carcass analysis, there was a strong sex predilection towards females amongst the social herd ungulates such as zebras and wildebeest, but this probably reflects the natural sex bias in these breeding herds. There was no sex bias amongst the white rhinoceroses. All carcasses seen were those of adults or young adults. The carcasses showed classical macroscopic liver lesions, widespread haemorrhages and pulmonary oedema. Histopathology of the liver was typical of classic microcystin poisoning, which has been described as causing fulminant liver necrosis and disruption of the hepatic architecture followed by liver failure with coagulopathy (Hooser 2000).

The fact that with the exception of wildebeest very few other ruminant species were affected may reflect some protective mechanism as a result of ruminal dilution or breakdown of cyanotoxins by the rumen micro-flora (Manubolu *et al*. 2014). White rhinoceroses and zebras, which seemed particularly commonly affected, are both hindgut fermenters and the few wildlife cyanobacterial intoxications reported previously included another event involving white rhinoceroses (Soll & Williams 1985).

Several options were considered to address these cyanobacterial intoxication events, but because of the bio-diverse nature of the large mammalian and reptilian species in this ecosystem, few conventional approaches for dealing with these blooms appeared appropriate or practical. The use of algacides such as copper sulphate was the first treatment option considered, but it proved impractical because of large releases of toxin from the dying algal cells, which meant that the dam would then have to be fenced off for at least 6 weeks (Nchabeleng *et al*. 2014). Phosphate binders that combine with dissolved phosphates and cause these nutrients to sediment into the bottom sludge was another option. However, they would be unlikely to succeed in a situation that includes the sediment-stirring activities of resident hippopotami, other large wading terrestrial species and bottom-feeding fish.

The use of floating booms or curtain devices on the down-wind side of the dam were deemed impractical because of the frequent changes in wind direction, as well as the physical presence of resident hippopotami and crocodiles, as well as wading elephants and buffaloes. Exclosure fencing of the dams was deemed impractical because of the high densities of elephant, hippopotami and rhinoceroses in the area. Controlling the hippopotamus populations was seriously considered and was practically feasible. It was reasoned that the disturbance factor caused by the initial hippopotamus control efforts (shooting) would encourage the remainder of the hippos to disperse to other aquatic environments. However, this would not address the huge amounts of accumulated nutrient-rich sediment in these impoundments.

The option of building spillways to assist in flushing these systems was considered but the process would unfortunately not remove the bulk of nutrients in the sediment. The possibility of building in a large bore sluice drain with a manually controllable valve was considered but was rejected because of the rate of ‘silting up’ experienced with these impoundments.

A *laissez faire* option of doing nothing was not considered because of the concern over the white rhinoceros losses and the threat to other rare or endangered species caused indirectly by these man-made structures.

## Conclusion

Eutrophication and harmful cyanobacterial blooms are becoming a dominant feature in still water impoundments across the globe. The events described in the KNP were both temporally and spatially clustered, and it would appear that the building of earthen dams across seasonal drainages with small catchments, together with increased hippopotamus densities played an important role in sediment trapping and increased rates of eutrophication of these water bodies. In conclusion, the decision to sacrifice these particular dams was rapidly effective and successful. It is important to appreciate that future responses to problem dams need to balance both the tourism and conservation value of the waterholes and dams. This may involve engineering solutions to reconstruct dams as artificial wetlands with a diversity of water depths and submerged, floating and in-stream vegetation to drive the system into and maintain it in a plant dominated, clear water state.

Finally, many conservation areas in relatively arid savannahs are dependent on boreholes, natural pans and storage dams to supply and stabilise surface water. Wildlife managers should be aware of and alert to the possibility of cyanobacterial blooms, when multispecies mortalities occur and are clustered near a water point.
